# Supplementary figures and images for: Data-driven insights into neighborhood adherence to cancer prevention guidelines in Philadelphia
Source: PLoS One. 2024 Nov 20;19(11):e0313334. doi: 10.1371/journal.pone.0313334 (PMC11578512; doi:10.1371/journal.pone.0313334)

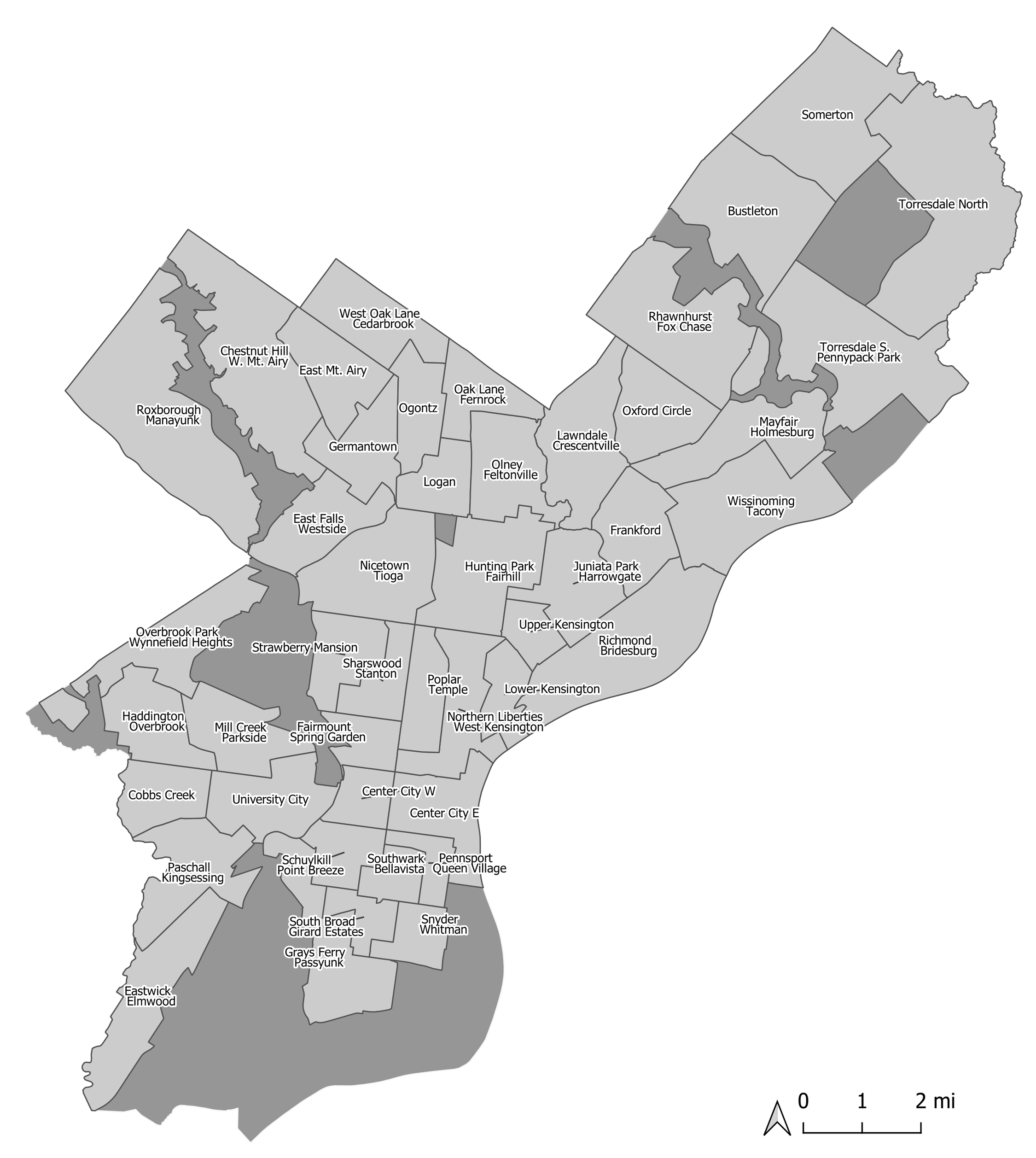

Supplement: S1 Fig — (TIF) [file pone.0313334.s001.tif]

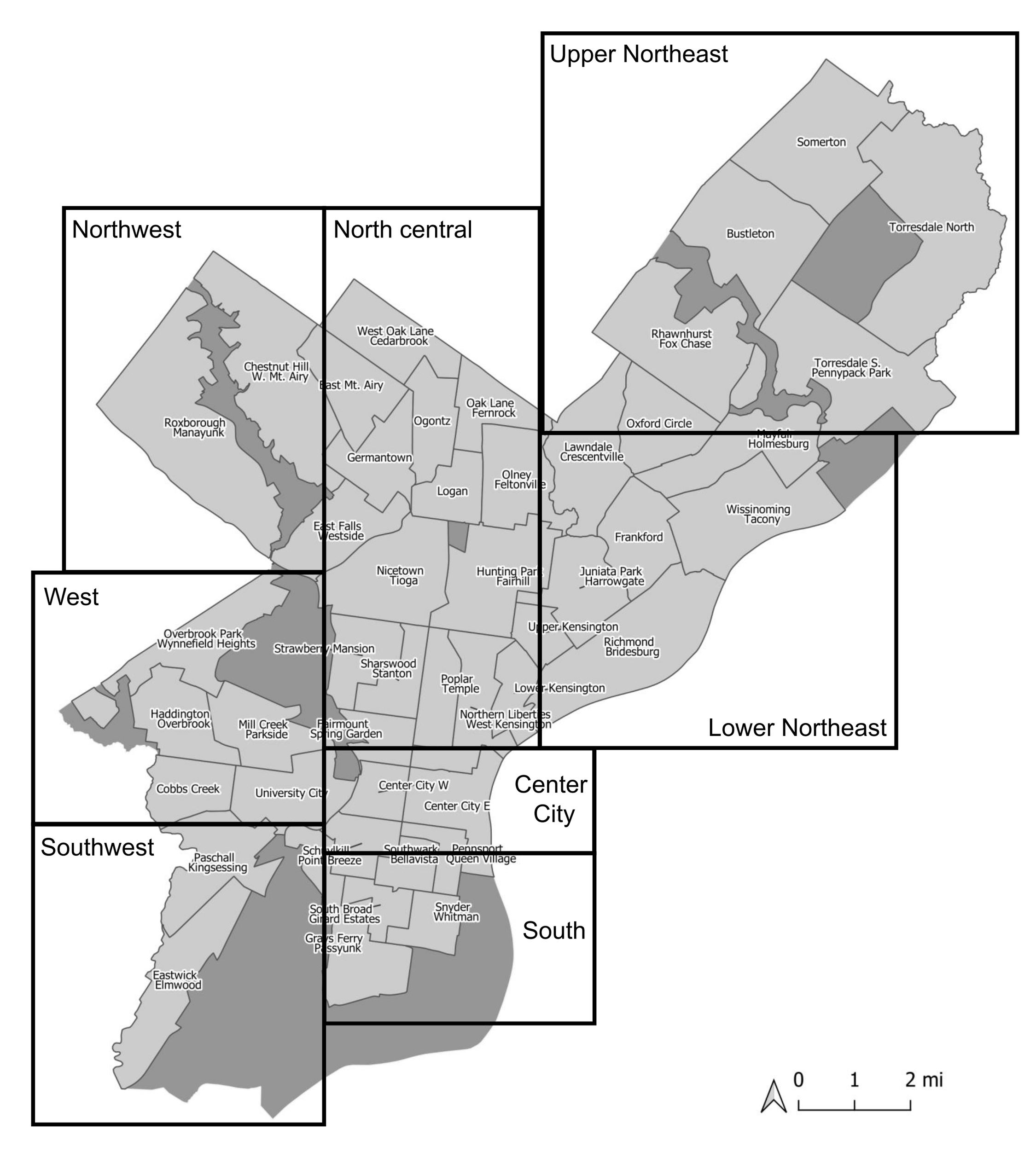

Supplement: S2 Fig — Neighborhood map (matching S1 Fig) that includes rectangles around the different regions of the city. (TIF) [file pone.0313334.s002.tif]

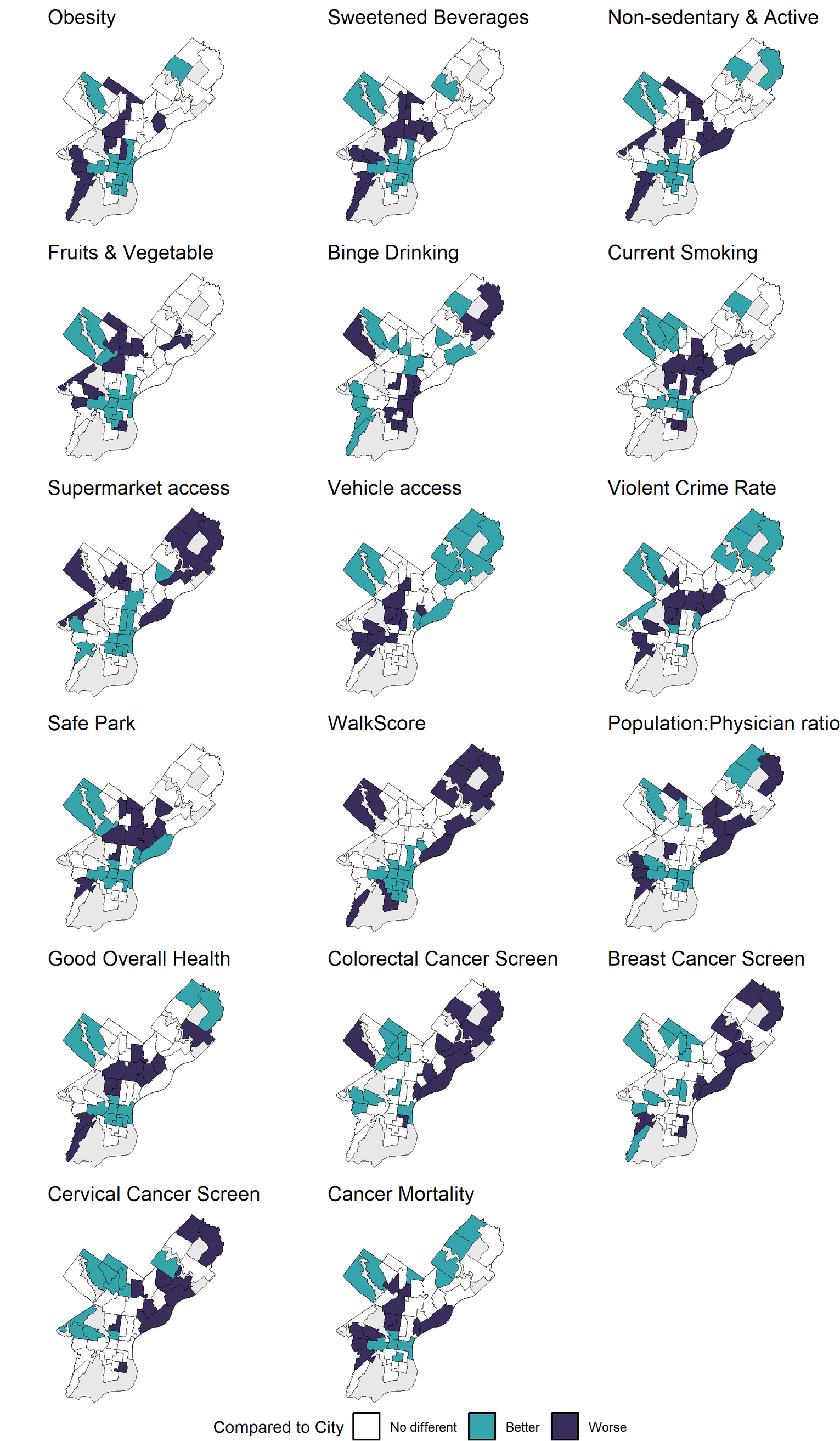

Supplement: S3 Fig — Series of small maps showing the neighborhood-level “better,” “worse,” and “no different” categories for each of the 18 measures included in the indices and for cancer mortality. (TIF) [file pone.0313334.s003.tif]
