# Supplementary material for: Data-driven insights into neighborhood adherence to cancer prevention guidelines in Philadelphia
Source: PLoS One. 2024 Nov 20;19(11):e0313334. doi: 10.1371/journal.pone.0313334 (PMC11578512; doi:10.1371/journal.pone.0313334)
Supplement: S1 Table — Table detailing each recommendation, where it originated, the measure(s) used to measure adherence, the data sources and years for each measure, the technique for aggregating data to the neighborhood-level, and the direction (whether being above the city overall for that measure is considered better or worse). (DOCX) [file pone.0313334.s004.docx]

**S1 Table: Detailed information about included measures.**

| **Recommendation** | **Target Established By** | **Measure(s)** | **Data Source** | **Data Year** | **Method of Aggregation/Conversion to Neighborhood-Level Measure** | **Direction** |
| --- | --- | --- | --- | --- | --- | --- |
| #1- Achieve and Maintain a Healthy Weight Throughout Life | ACS Guidelines | The percent of Philadelphians with a BMI greater than or equal to 30 kg/m2 | SEPAHHS* | 2018 | Individual-level survey responses were aggregated to the CT**-level by PHMC.*** For the present analysis, SEPHAAS data were modeled at the neighborhood level using Bayesian statistical methods to generate smoothed prevalence estimates[1] age-standardized to the 2014-2018 population using Census ACS data | Above = Worse |
| #2- Be Physically Active | ACS Guidelines | The percent of Philadelphians exercising 30 minutes at least 3 times/week AND meeting the non-sedentary guidelines | SEPAHHS | 2018 |  | Above = Better |
| #3- Eat a Healthy Diet, with an Emphasis on Plant Foods | ACS Guidelines | Percent of Philadelphians consuming 5+servings of fruit/veggies daily | SEPAHHS | 2018 |  | Above = Better |
|  |  | The percent of Philadelphians who drank 1 or more sugar-sweetened beverages per day | SEPAHHS | 2018 |  | Above = Worse |
| #4- Limit Intake of Alcohol | ACS Guidelines | Percent of Philadelphians who are binge drinking (4+ drinks for women or 5+ drinks for men in two hours) in last 30 days | SEPAHHS | 2018 |  | Above = Worse |
| #5- Stay Away from Tobacco | Healthy People 2030 | Percent of Philadelphians that are current smokers | SEPAHHS | 2018 |  | Above = Worse |
| #6- Increase access to affordable, nutritious foods | ACS Guidelines | The percent of Philadelphians who are further than 1/2 mile from the closest supermarket | USDA Food Access Research Atlas | 2015 | A prevalence estimate produced by aggregating CT estimates weighted by the residential population contributed to the neighborhood by each CT | Above = Better |
| #7- Provide safe, enjoyable, and accessible environments for physical activity in schools and workplaces, and for transportation and recreation in communities | ACS Guidelines | Percent of households which own at least one vehicle | ACS | 2018 | Census data related to vehicle ownership was provided as counts at the CT level and was aggregated to the neighborhood level before normalizing by the relevant population to produce a neighborhood prevalence rate. | Above = Better |
|  |  | The average walk score (out of 100) based on intersection density, residential density, and accessibility of amenities such as grocery stores, parks, and restaurants | Walk Score TM | 2018 | A prevalence estimate produced by aggregating CT estimates weighted by the land area contributed to the neighborhood by each CT | Above = Better |
|  |  | The number of violent crimes (murder/homicide, aggravated assault, robbery, and rape) divided by the Philadelphia Population and transformed to a rate per 100,000 people | Philadelphia Police Department | 2017 | Calculated as a count of crime events within the neighborhood normalized by the residential population to generate a mean prevalence rate. | Above = Worse |
|  |  | The percent of Philadelphians who responded "yes" to having a park in their neighborhood that they felt comfortable visiting | SEPAHHS | 2018 | Individual-level survey responses were aggregated to the CT-level by PHMC. For the present analysis, SEPHAAS data were modeled at the neighborhood level using Bayesian statistical methods to generate smoothed prevalence estimates age-standardized to the 2014-2018 population using Census ACS data | Above = Better |
| Health-specific #1: Increase the proportion of adults who self-report good or better health | Healthy People 2030 | The percent of Philadelphians who self-reported a health status of good or higher | SEPAHHS | 2018 |  | Above = Better |
| Health-Specific #2: Provide population-based primary prevention services | Healthy People 2030 | Population per primary care provider (ie: number of persons per provider) | Close to Home report | 2016 | A prevalence estimate produced by aggregating CT estimates weighted by the residential population contributed to the neighborhood by each CT | Above = Better |
| Health-specific #3: Screening | Healthy People 2030 | The percent recommended based on age who received a colonoscopy within the past 10 years | SEPAHHS | 2018 | Individual-level survey responses were aggregated to the CT-level by PHMC. For the present analysis, SEPHAAS data were modeled at the neighborhood level using Bayesian statistical methods to generate smoothed prevalence estimates age-standardized to the 2014-2018 population using Census ACS data | Above = Better |
|  |  | The percent recommended based on age/sex who received a mammogram within the past 2 years | SEPAHHS | 2018 |  |  |
|  |  | The percent recommended based on age/sex who received a pap smear test within the past 3 years | SEPAHHS | 2018 |  |  |
| Overall Cancer Mortality | Not applicable | The total number of cancer deaths in Philadelphia divided by the total population | PA State Cancer Registry | 2012-2016 | Residential addresses of all cancer mortality data were geocoded to the census tract using ESRI Business Analyst 2016 Geocoder. Only records that geocoded to Philadelphia census tracts were included in the analysis. Age-specific mortality rates were calculated using five-year CT population estimates for each demographic sub-group from Census ACS such that the survey mid-point corresponds to the year of mortality. Population and cancer mortality counts at the CT-level were aggregated to the neighborhood-level before generating neighborhood mortality rates. | Above = Worse |

* SEPAHHS = Southeastern Pennsylvania Household Health Survey

** CT = Census tract

*** PHMC = Public Health Management Corporation

**References**

1. Quick H, Terloyeva D, Wu Y, Moore K, Diez Roux AV. Trends in Tract-Level Prevalence of Obesity in Philadelphia by Race-Ethnicity, Space, and Time. Epidemiology. 2020;31: 15–21. doi:10.1097/EDE.0000000000001118
