# Supplementary material for: Data-driven insights into neighborhood adherence to cancer prevention guidelines in Philadelphia
Source: PLoS One. 2024 Nov 20;19(11):e0313334. doi: 10.1371/journal.pone.0313334 (PMC11578512; doi:10.1371/journal.pone.0313334)
Supplement: S2 Table — The results of two univariate regressions predicting mortality. Model 1 uses the final ACS Physical Activity, Nutrition, and Smoking Guidelines Index to predict cancer mortality rates at the neighborhood level in a linear regression. Model 2 does the same using the final Preventive Services Guideline Index. Both models are highly significant, with p-values of under 0.001 and adjusted R squares of 0.45 and 0.22, respectively. (DOCX) [file pone.0313334.s005.docx]

**S2 Table: Univariate regressions predicting cancer mortality with each adherence index**

| **Model 1** | |
| --- | --- |
| Outcome Measure: Cancer Mortality | |
| Intercept | 196.0 (3.62)*** |
| ACS Physical Activity, Nutrition, and Smoking Guidelines Index | -22.3 (3.66)*** |
| Adjusted R^2^ | 0.45 |
| No. Observations | 46 |

***p<0.001; **p<0.01; *p<0.05

| **Model 2** | |
| --- | --- |
| Outcome Measure: Cancer Mortality | |
| Intercept | 196.0 (4.30)*** |
| Preventive Services Guideline Index | -16.01 (4.35)*** |
| Adjusted R^2^ | 0.22 |
| No. Observations | 46 |
| ***p<0.001; **p<0.01; *p<0.05 |  |
