# Supplementary material for: Data-driven insights into neighborhood adherence to cancer prevention guidelines in Philadelphia
Source: PLoS One. 2024 Nov 20;19(11):e0313334. doi: 10.1371/journal.pone.0313334 (PMC11578512; doi:10.1371/journal.pone.0313334)
Supplement: Attachment — Submitted filename: PLOSOne_Human_Subjects_Research_Checklist_SL.docx [file pone.0313334.s006.docx]

**Human Participants Research Checklist**

***Complete the following if your study involved human participants or human participants’ data. These questions should be addressed for prospective and retrospective studies.***

1. Did you obtain ethics approval for this study?
   - If yes, please upload (file type “Other”) the original approval document you received from your ethics committee. If the original document is in another language, please also provide an English translation.

_X__ Uploaded	_ __ N/A

- If you did not obtain ethical approval, please explain why this was not required below.

**Explanation:** This study utilized secondary data sources from primarily publically available data. Our IRB reviewed this study and deemed it as “exempt”.

2. If you prospectively recruited human participants for the study – for example, you conducted a clinical trial, distributed questionnaires, or obtained tissues, data or samples for the purposes of this study, please report in the Methods:
   i. the day, month and year of the **start and end** of the recruitment period for this study.
   ii. whether participants provided informed consent, and if so, what type was obtained (for instance, written or verbal, and if verbal, how it was documented and witnessed). If your study included minors, state whether you obtained consent from parents or guardians. If the need for consent was waived by the ethics committee, please include this information.

___ Completed	__X_ N/A.

**Explanation**: A waiver of consent was granted from the IRB given we are working with secondary data sources, many of which are publically available.

3. If you are reporting a retrospective study of medical records or archived samples, please report in the Methods section:
i. the day, month and year when the data were accessed for research purposes
ii. whether authors had access to information that could identify individual participants during or after data collection

___ Completed	_X__ N/A

**Explanation:** The dates associated with secondary data sources used in this study are provided in the manuscript and/or supplement. We did NOT Have access to individual, identifiable data for this study.
