# Supplementary material for: Data-driven insights into neighborhood adherence to cancer prevention guidelines in Philadelphia
Source: PLoS One. 2024 Nov 20;19(11):e0313334. doi: 10.1371/journal.pone.0313334 (PMC11578512; doi:10.1371/journal.pone.0313334)
Supplement: Attachment — Submitted filename: Response to Reviewers.docx [file pone.0313334.s007.docx]

Response to reviewer comments on PLOS One submission:

“Data-driven insights into neighborhood adherence to

cancer prevention guidelines in Philadelphia”

**Comments to the Author**

**Reviewer #1: Thank you for allowing me to review this interesting study. This study proposes to address the important gap of generating neighborhood-level cancer-related guideline adherence measures and indices in Philadelphia PA as an example for tracking cancer risk factors. While the question is important, the analytic choices could be improved or further justified. Most importantly, the choice of cut-off for assigning numeric value within each measure could influence final index creation substantially, and differently for each measure leading to inherent down-/up-weighting. This and more minor comments follow.**

Thank you for reviewing our manuscript and for your thoughtful suggestions. We fully agree that the changes you have suggested would strengthen the research and have applied as many of them as possible. Most substantially, we have re-run our analysis to assign the cut-off for each measure using the same method (quartile breaks) so as to make the weighting of each measure uniform within each recommendation and improve interpretability of our findings and the usability of the index. Please accept our sincere appreciation for the time and consideration you have contributed to supporting this research through your thorough review. Our manuscript has significantly improved.

**Introduction
1. Lines 60-62. ‘nearly 20% of all new cancers diagnosed are attributable to modifiable risks’, the reference supporting this cannot be found and 20% is lower than many other published estimates (Islami et al CA a cancer journal for clinicians 2018, GBD 2019 Cancer Risk Factors Team Lancet 2022, Collatuzzo and Boffetta Ann Rev Pub Health 2023). Consider revising.**

Thank you so much for catching this and suggesting the Islami reference. Our original reference was from the AACR Cancer Progress Report, but it did not include smoking, rather, it only included excess body weight, alcohol intake, poor diet, and physical activity. The reference you provided is much better given the topic of this analysis. We have updated the introduction (lines 74-76) to cite Islami et al. and state: “However, approximately 45% of cancer deaths are attributable to modifiable risks, including smoking, excess body weight, alcohol intake, poor diet, and lack of physical activity, which if addressed, could improve cancer prevention(3).”

**Methods
3. Cancer mortality rates from 2012-2016. It is unclear why the ‘outcome’ cancer data is dated prior to the ‘exposures’ when cancer data presumably exists post-2018 or most exposure data pre-2012.**

Thank you for raising this point. We agree that a better alignment of the risk factors and cancer data would strengthen this analysis. However, we were limited by the data we could access for this analysis. Our indices combine multiple years to speak generally about adherence at the neighborhood level and we use the mortality data with some overlapping years to show an example of how comparing the index to an outcome measure could be helpful to inform public health approaches. The purpose of the development of these indices is to help identify neighborhoods to prioritize for intervention, and not to predict cancer outcomes. To further address your concern, we also added the following to the limitations section of the manuscript. Lines 509-515 read as:

“A third limitation is that we use available data and years ranges; as a result, when we compare our indices to cancer rates, the year ranges only overlap slightly at the beginning of that time frame. However, the primary purpose of the manuscript was to create an index that is reflective of modifiable risk factors over time and preliminarily test the utility of the measure by comparing poor adherence to a disease measure. Future studies should take into consideration data availability and the time frame of exposures and outcome.”

**4. Creation of indices. Given availability of cancer burden attributable risk estimates for many of these measures, it is unclear why each is given equal weight in the index creation; e.g., tobacco is well accepted to contribute substantially to cancer mortality, while >½ mile from a supermarket likely contributes far less to cancer mortality.**

This is a very intriguing suggestion and we would be interested in seeing this applied as a future direction or next step. Determining an appropriate weight for each measure is beyond the scope of this analysis, particularly given this is one of the first analyses to try to evaluate and compile a built environment score. At this point, justification of weights would be a challenge and potentially considered premature without proper expert opinions and systematic reviews, particularly because the weighted score would be based mostly on findings from studies of measures, like smoking at the individual level, as opposed to at the area-level. The intended purpose of our analysis is to inform where and which modifiable factors should be prioritized for intervention in high cancer mortality neighborhoods within a single city, not to predict cancer outcomes. To address your comment, we have added to the discussion the following on lines 522-531: … a limitation of this analysis is that all variables are weighted similarly, when it is possible some measures at the area-level (e.g. living in an area with high smoking rates) could have more of an impact on disease outcomes than others (e.g. living >1/2 mile from a supermarket). This impact could vary depending on the outcomes studied and in the context of individual level behaviors. Therefore, findings must be mindfully interpreted, and future cancer control and prevention studies to investigate risk factor differences by neighborhood using additional sophisticated statistical testing are suggested. This could include weighting of area-level data for the generation of adherence scores, as well as multi-level analyses to better understand how the neighborhood may influence cancer prevention guideline adherence on an individual-level.

**5. Creation of indices. Please provide justification for why sugar sweetened beverage consumption is part of body weight recommendation when it is not part of body weight in the original ACS recommendation (doi.org/10.3322/caac.21719), rather it’s part of diet.**

Thank you for catching this error. We re-ran our analysis with sugar sweetened beverages contributing to recommendation 3 on healthy diet rather than recommendation 1 on healthy weight. You will see the following changes in the revised manuscript:

1. In Table 1 and Supplemental Table 1, the rows containing information about this measure have been moved from recommendation 1 to recommendation 3.
2. Maps and Tables of results were updated to reflect the updated analysis
3. Paragraph 2 in the Results section (Lines 259-279) were modified to reflect this change.

**6. Creation of indices. I having a hard time understanding calculation of the vehicle access measure involving MOE. Are MOE’s of Philly and Philly neighborhoods being compared to one another? Or the ACS mean estimates Philly and Philly neighborhoods of vehicle access being compared to one another while accounting for MOEs to generate 95% CIs? If the latter, I’m still confused where z-scores come into the calculation and how the vast majority of neighborhoods appear to be outside the |1.96| range of the z-score. Can you please clarify?**

Thank you for raising this point of confusion. The MOEs originally used for the vehicle access measure were based on the MOEs provided from the Census. However, in line with the rest of the changes made to this manuscript, we now employ only the estimate (percent of households that own a car) by neighborhood and categorize neighborhoods based on quartiles.

**7. Creation of indices. I am concerned that Step 2 recommendation z-score, and therefore, step 3 overall adherence scores based on sums of step 2 z-scores is highly sensitive to step 1 choice of cut-off for better/worse/no different of each measure. For example some measures will generate a 1/better or -1/worse based on quartiles of the 46 neighborhoods; guaranteeing that the measure will be 1/better for ~12 neighborhoods and -1/worse for another ~12 neighborhoods. However, other measure’s assignment of 1/better or -1/worse is based on exceedance of 95% credible intervals (>97.5% or < 2.5%). Predictably, those credible interval-based measures yield far fewer 1s and -1s, and therefore, are essentially down-weighted in creation of the overall adherence scores. This should be addressed ideally through creation of measures that are not inherently weighted differently by assignment of values (1,0,-1), and/or sensitivity analyses with various cutoff values assigned to each measure and results reported in the supplement. Noting as a limitation in the Discussion is correct, but this limitation is addressable within the scope of this study’s purpose and created by the study design itself.**

Thank you for accurately pointing out that this is something we can address. Based on your concern, which we fully agree with, we have recalculated the indices to use quartile breaks for better/worse designations on each measure before rolling them into recommendations. As such, the following changes have been made to the manuscript:

1. We re-ran the analysis and produced all new figures reflecting our new results, which are mostly consistent with the previous results, but do show some variation
2. We updated our table 1 to show the quartile range of better and worse categories rather than the number of neighborhoods that fell into each
3. We removed the following paragraph, as it is no longer relevant because all measures have the same distribution: “Factors related to the built environment, such as access to a supermarket or primary care provider, have a quarter or more of the 46 neighborhoods at each extreme (recommendation #7). Conversely, measures focused on residents' risk behaviors, such as having a healthy diet, limiting alcohol intake, or completing recommended cancer screenings, have as few as zero (recommendations #3 & #4) and as many as 6 (recommendations #1 & #5) neighborhoods at an extreme (better/worse).”
4. After making these changes, our findings did remain similar with some minimal changes that are not noted in the limitations section the following statement lines 520 – 522, “Additionally, we have demonstrated a process for generating guideline adherence indices, however, various methods could be applied that may result in somewhat different neighborhoods being identified.”

**8. Figure 3. Why were maps limited to neighborhoods identified as high mortality and low/worse adherence, as opposed to calculating and/or displaying a correlation – spatial/LISA or aspatial/spearman - between the measures? If the goal is to use these indices for neighborhood-level tracking of cancer related risk factors, then it would be important to know if they correlate with neighborhood level cancer burden. If they do not, then their utility seems diminished.**

We appreciate and agree with this suggestion, in that, if the indices were not correlated with cancer burden, then either the ACS and HP have done a poor job of creating relevant cancer prevention guidelines (highly unlikely) or we have produced – either through poor measures or poor calculations- an index that cannot be considered a measure of adherence to the guidelines (possible). Therefore, to speak to the validity of the indices, we agree it is important to show that they are indeed correlated or even associated with cancer burden. We updated the manuscript to reflect this below.

Although we include additional statistical analyses, we want to be careful about guiding readers on the utility of the generated indices. We are not measuring individual risks. Therefore, we want to make sure we clarify that these indices are not meant to predict cancer burden, but rather to guide and inform targeted interventions in neighborhoods and regions of Philadelphia where we see that cancer burden is high and adherence to modifiable factors related to cancer prevention guidelines is low. As such, the following edits have been made to the manuscript:

1. We conducted a spearman correlation test and, upon finding that each index is significantly correlated with cancer mortality, we conducted a linear regression to determine the extent to which variation in cancer mortality between neighborhoods can be explained by each index.
   a. We introduced this in the methods, lines 233 –235, “To establish whether the resulting indices are statistically related to cancer burden, a Spearman test of correlation was first conducted before testing for association using a series of univariate linear regressions (S2 Table).”
   b. Mentioned it in the results, lines 332 – 335, “Both indices were correlated with cancer mortality and a linear regression found that 44.5% of between-neighborhood variation in cancer mortality can be explained by the ACS Physical Activity, Nutrition, and Smoking index alone and 21.8% can be explained by the HP Prevention Services Guidelines.”
   c. Mentioned in the discussion that this 44.5% seems comparable to the literature suggesting that 45% of cancer deaths are estimated to result from modifiable risk factors in the US, lines 338-341, “As prior research suggests that 45% of cancer deaths are attributable to modifiable factors(3), it is noteworthy that the ACS Physical Activity, Nutrition, and Smoking index can statistically explain the same amount of variation of cancer mortality between neighborhoods utilizing area-level data.”
   d. Provided the regression tables in the supplemental materials (S2 Table)
2. Clarify the intended use of the indices in multiple places.
   a. In the last line of the methods, lines 236-238, we now state: “Finally, focusing on neighborhoods where high mortality and poor adherence co-occur, recommendations responsible for the poor adherence scores were identified to suggest possible points of intervention specific to a given neighborhood.”
   b. In the results, lines 335-338, we now state: “While the purpose of these indices is not for predicting cancer burden, as they focus only on modifiable behavioral and built environment factors at the area level, this association is important to establish as it speaks to the utility of the indices.”
   c. Mention in the discussion, lines 515 - 531 that we found an association between the indices and cancer mortality, but that this is not meant to be predictive as we are using aggregated area-level measures. We also suggest that future studies conduct multi-level analysis including individual and area-level data:

“Lastly, this is not a causal study and the indices created herein are not intended to predict cancer outcomes. Rather, we use area-level measures and summaries to help inform cancer prevention efforts. While we find co-occurrence of high cancer mortality and low adherence in some neighborhoods and find a significant association between the two, we cannot assert that low adherence to a particular recommendation is causing a high cancer mortality in a given location…. Therefore, findings must be mindfully interpreted, and future cancer control and prevention studies to investigate risk factor differences by neighborhood using additional sophisticated statistical testing are suggested. This could include weighting of area-level data for the generation of adherence scores, as well as multi-level analyses to better understand how the neighborhood may influence cancer prevention guideline adherence on an individual-level.

**Results
9. Temporal mismatch and causal language. Related to comment 3 above, relationships between the temporally mismatched variables are described using ‘coincided’, ‘driver’, ‘influencing’. Consider strengthening methods – temporally aligned data, causal inference, etc. - to justify causal language or describe relationships more appropriately.**

Thank you for picking up on this. Although we had mentioned in the limitations that this study is not causal, we agree that our language in the paper suggests otherwise. We have revised our language throughout to be more sensitive to this nuance, and we tempered our language. For example, the following changes were made:

1. Line 23, we changed the wording from “suggesting a possible driver of” to “suggest possible points of intervention”
2. In line 536-537 in the Discussion, we changed the wording about the built environment from “this study’s findings highlight the importance of the built environment in influencing guideline adherence” to “this study’s findings suggest that the built environment may relate to guideline adherence.”
3. Revised the last sentence in the results section of the abstract to avoid saying driver or coincided. It now states: “In neighborhoods where poor guideline adherence overlapped with high cancer mortality, the built environment emerged as a potentially important factor.
4. In line 325, changed heading to clarify that we are looking at recommendations that drive poor adherence, not that drive cancer mortality: “Identifying Recommendations Related to Poor Guideline Adherence in High Cancer Mortality Neighborhoods”

**10. S3 10 min walk to park. As a quartile measure, how are there no better/quartile 4 neighborhoods?**

Thank you so much for pointing that out. Upon further investigation, this measure does not have sufficient variation between neighborhoods to be broken into quartiles, which is why you correctly note that we did not have any “better” neighborhoods. This measure was based off a national dataset that shows insufficient variation within a single city. As such, we have removed this measure from our index. However, we have referenced the dataset in our discussion, as we find it pertinent to discuss that while much of Philadelphia is in close proximity to a park, a smaller proportion of residents feel they have a safe park that they feel comfortable visiting within walking distance. This ties into our discussion of how crime may be influencing cancer prevention guideline adherence in Philadelphia neighborhoods. Please note the following changes:

1. The “The percent of urban population within a 10-minute walk to a park’ measure was removed from Table 1. ACS Physical Activity, Nutrition and Smoking Guidelines, as we reran the analysis to exclude that measure in recommendation 7.
2. ParkScore™ was removed from line 264 where we describe the datasets included in recommendations 6 and 7. The remainder of that paragraph was edited to describe that we use 4 measures for recommendation 7 (rather than our original 5). The following was deleted from this paragraph, but relocated to the discussion (lines 477 - 478) with a citation of the ParkServe data: “Philadelphia residents have a high access to parks within a 10-minute walk from their home (95% vs. 55% nationally).”
3. The row containing ParkServe data in Supplemental Table 1 was removed and in the paragraph starting on line 160, which references S1 Table, the text “and ParkServe data (2019) provided by the Trust for Public Land” was removed.
4. Rather than mentioning our own examination of park availability, we updated Paragraph 7 of the discussion (lines 476-480) to state: “The spatial overlap between crime and other measures of guideline adherence included in this study warrant additional consideration. For example, while Philadelphia residents have a high access to parks within a 10-minute walk from their home (95% vs. 55% nationally(34,35)), only 75.8% have a park they feel safe visiting within a 15-minute walk from their home. The difference between park availability and comfort visiting parks (95% vs 76%) is notable.”
